# Preparation and Thermal Properties of Modified Cu_2_O/Polypropylene (PP) Composite

**DOI:** 10.3390/ma13020309

**Published:** 2020-01-09

**Authors:** Yurong Wu, Longshan Xu, Yanying Jiang

**Affiliations:** 1 School of Materials Science and Engineering, Xiamen University of Technology, Xiamen 361024, China; winwyr@126.com; 2 School of Information and Communication, Guilin University of Electronic Technology, Guilin 541000, China; weihuigs@gmail.com

**Keywords:** superfine Cu_2_O sphere, modified Cu_2_O, Cu_2_O/Polypropylene composite, KH570, thermal properties

## Abstract

A uniform, monodispersed superfine cuprous oxide (Cu_2_O) sphere with a mean diameter of 850 nm has been synthesized by solution reduction. The study reported the synthesis and thermal properties of Cu_2_O/PP composites for the first time. The surface modification of the superfine Cu_2_O sphere was carried out by using a silane coupling agent KH-570. Fourier-transform infrared (FTIR) spectroscopy and the thermogravimetric analysis (TGA) curve revealed that the Cu_2_O had been successfully modified by silane coupling agent KH570. The scanning electron microscope (SEM) shows that the modified Cu_2_O can be uniformly dispersed in the polypropylene (PP) matrix, because through surface modification, there are some active functional groups on its surface, such as the ester group, which improves its compatibility with the PP matrix. The thermal stability of Cu_2_O/PP composites was improved by adding a small amount of Cu_2_O (1 wt % of PP). Therefore, based on the potential bacteriostasis of cuprous oxide, the low cost of PP and the results of this study, it is predicted that Cu_2_O/PP composites can be used in infant preparation (such as milk bottles) with low cost and good thermal stability in the near future.

## 1. Introduction

Polypropylene (PP), as a general-purpose plastic, has the advantages of excellent physical properties and easy molding and processing, plays an increasingly important role in the plastics industry, and is widely used in various fields such as chemical, machinery, automobile, food packaging, infant products, etc. [1,2,3,4]. However, PP is a non-polar polymer with poor toughness, high temperature brittleness and large molding shrinkage, which limits the engineering application of PP. How to make plastics and the special functions of PP engineering and to expand its use is an important direction for PP development. Thus, PP is usually modified through being blended with other resins and filled with the introduction of inorganic fillers, such as calcium carbonate [5,6], metallic powder [7,8] and nano-carbon (carbon tube, graphene) [9,10,11,12]. So polypropylene-based nano-composites provide a new way for polypropylene modification. For example, only a small number of nanoparticles can improve significantly the properties of polymers, such as electrical properties [13,14], mechanical properties [15,16] and thermal properties [12,16,17,18,19]. Bafana A P et al. [12] reported that graphene/polypropylene nanocomposites were prepared using the solution mixing method, and it was found that only a small amount of graphene can improve the thermal properties of PP. Jisheng Ma et al. [19] synthesized Polypropylene/clay (PP/clay) nanocomposites via intercalative polymerization. The transmission electron microscope (TEM) image showed that the clay was exfoliated into nanometer size and dispersed uniformly in the PP matrix, and the thermal stability of the nanocomposites significantly increased. 

Therefore, adding nano/micro materials to the PP matrix to improve their performance has become a hot research topic. However, as we all know, when particle size is decreased to the nanoscale range, its properties will significantly change. Among nanoparticles (NPs), metal oxide NPs (i.e., ZnO, ZrO_2_, SiO_2_, Fe_3_O_4_, CuO and etc.) are attracting considerable interest due to their unique physical and chemical properties [20]. Their unique properties of metal oxide NPs strongly depend upon NPs preparation and dimension [21,22]. However, the main problem in NPs application is the high tendency of metal oxide NPs to adhesion and aggregation. Surface modification of the metal oxide NPs with organic compounds has been employed to overcome the problem and reduce the particle surface energy and decrease their tendency to agglomerate [23,24]. In addition, due to their large specific area, inorganic nanoparticles are easy to agglomerate, and are not easily dispersed in the polymer matrix, which limits their reinforcing effect. Therefore, in order to solve this problem, inorganic nanoparticles are often modified [25,26,27]. Xia et al. [26] have used modified ZrO_2_ NPs as an additive. To improve the dispersion and interaction of the ZrO_2_ with the epoxy coating, the surface of NPs was modified by a styrene coupling agent by the post modification. Based on the above description, the surface of NPs plays an important role in the properties of NPs, counting the dispersity, solubility, stability, reactivity, melting point and electronic structure. Mallakpour et al review the synthesis, properties, applications and surface modification of metal oxide NPs, the classification of coupling agents, and the interaction between metal oxide NPs and coupling agents [28]. Therefore, in this study we reported the surface modification of metal oxide NPs cuprous oxide with silane coupling.

Cuprous oxide (Cu_2_O) is a rare semiconductor material that can be excited by visible light with a band gap of about 2.0 eV. Cu_2_O has nontoxic and low preparation cost, can directly degrade organic matter by sunlight without secondary pollution, and has high theoretical utilization efficiency. Therefore, it is one of the green catalysts with great development prospects. Up to now, cuprous oxide has been prepared by several different methods, such as electrodeposition, solvothermal/hydrothermal methods, sonar–chemical methods, γ-Irradiation, the liquid-phase reduction, etc. [29,30,31,32]. Based on the superior properties of Cu_2_O, it has many potential applications in solar energy conversion [33], photocatalytic degradation [34,35], marine antifouling coating [36], lithium ion batteries [37], carbon dioxide [38], gas sensors [39], and so on.

The incorporation of inorganic nanoparticles in polymer has been found to improve the thermal [16,18,19], mechanical [15,16,17], electrical [14], corrosion resistance [25,26] and antimicrobial properties of the polymer [21,35,40,41]. The improvement of the inorganic nanocomposite properties depends on filler type, size, shape, the degree of dispersion of the NPs in the polymer matrix and the degree of adhesion of NPs with polymer chains [42,43]. Generally, there are two methods by which inorganic NPs are dispersed/incorporated in polymeric materials, either as additives by direct mixing with polymer [12,40,41,42], or as reactive materials by in situ polymerization [16,19]. To date, there are few studies on Cu_2_O/PP composites. Yongqian Shi et al. [40] reported that the thermal and smoke suppression properties of CuO/PP and graphene/PP. The thermal and smoke suppression properties of CuO/PP and graphene/PP were efficiently improved, compared with native PP. Palza et al. [41,42] prepared copper polypropylene-based composites by melting blend. After 4 h these composites, with 1 *v*/*v* % of copper nanoparticles, kill > 99.9% of *Escherichia coli* (*E. coli*). In those above studies, they focused on the thermal property, smoke suppression property and antibacterial activity of copper–polypropylene composites. To our knowledge, the study of Cu_2_O/PP composite was fabricated for the first time. Therefore, the development of Cu_2_O/PP composite material with both antibacterial properties and heat resistance is essential. The synthesized Cu_2_O/PP composites are expected to be used in infant products. In this present work, a superfine Cu_2_O sphere was synthesized by solution reduction, and modified Cu_2_O/PP composite materials were synthesized by melting blend. The modification of cuprous oxide is analyzed by an infrared spectrum. Dispersion of modified Cu_2_O in PP was characterized by SEM. The thermal stability of Cu_2_O/PP and modified Cu_2_O/PP composite materials was investigated by TGA and Differential scanning calorimetry (DSC).

## 2. Experimental Section

### 2.1. Raw Materials

CuSO_4_·5H_2_O (≥ 99.7%, Analytical Reagent, AR), NaOH (≥ 99.7%, AR), polyethylene glycol (PEG, ≥99.7%, Chemically Pure, CP), and glucose were purchased from XiLong Scientific Co. Ltd. (Shantou, China). Gelatin was supplied from Shanghai Jinsui Biotechnology Co. Ltd (Shanghai, China). Food grade PP-H masterbatch was purchased from Sinopec Beihai Refining and Chemical Co. Ltd. (Beihai, China), the silane coupling agent KH-570 (98%) was purchased from Sinopharm Chemical Reagent Co. Ltd. (Shanghai, China), xylene (AR) was supplied by Xilong Science Co. Ltd. (Shantou, China), while isopropanol (AR) was supplied by Sinopharm Chemical Reagent Co. Ltd. (Shanghai, China), and benzoyl peroxide (AR) was purchased from the Taizhou yateng Chemical Materials Co. Ltd. (Taizhou, China). De-ionized water (DI) was obtained from our laboratory itself. All reagents were used without further purification.

### 2.2. Synthesized of Superfine Cu_2_O Sphere

The Cu_2_O sphere was prepared by the following procedure. First, solutions of 1 g gelatin in 100 mL deionized water were prepared, and then sonicated for 30 min, and labeled as A solution. A set of solutions were prepared by adding 0.2 mol/L copper sulfate pentahydrate (Cu(SO_4_)_2_·5H_2_O) to 100 mL deionized water, in which a certain amount of polyethylene glycol (PEG) as surfactant, was labeled as the B solution. Secondly, B solutions were added to A solutions drop by drop, and then heated at 50 °C in a water bath and stirred continuously. Third, 100 mL 0.1 mol/L aqueous glucose solution was added drop by drop, and then 100 mL 3 mol/L NaOH solution was also added drop by drop. The mixture was kept at 50 °C for another 30 min. Magnetic stirring was continuously applied throughout the entire process of reduction and particle growth. The Cu_2_O particles were separated from the solution by centrifugation at 2000 rpm for 5 min, and was washed several times by deionized water and ethanol. The resultant product was dried at 60 °C in a vacuum oven for 6 h.

### 2.3. Modification of Cu_2_O

First, 1 g dried cuprous oxide was dispersed into 50 mL of distilled water, and the mixture was magnetically stirred for 30 min. Second, 10 mL isopropanol was added into the stirring solution. Third, a certain amount of KH570 was added to the stirring solution, the mixture was stirred for 1 h and then sonicated for 30 min. The suspension was centrifuged, washed by distilled water and absolute ethanol for several times to remove the remaining KH570, and dried in a vacuum oven at 80 °C for 4 h. The product was labeled as M-Cu_2_O. Additionally, the unmodified Cu_2_O is labeled as 0-Cu_2_O.

### 2.4. Synthesis of Cu_2_O/PP Composite

Cu_2_O/PP composite was synthesized using melt blending [39,40,41]. 10 g polypropylene (PP) and an appropriate amount of xylene were added to the three-necked flask, heated in an oil bath and stirred until completely dissolved. At the same time, a certain amount of Cu_2_O (1 wt % of PP) was added to an appropriate amount of xylene solution, and dispersed ultrasonically for 30 min. After the PP is completely dissolved, the ultrasonicated Cu_2_O solution was added to the PP xylene solution, and stirred 15 min, and then the initiator benzoyl peroxide xylene solution was added to the mixture. During the above reaction, the mixture was heated at 130 °C for 3 h. Then stopping heating, the system was cooled to 70 °C, the reactant was taken out, soaked in absolute ethanol for several times, and dried to constant weight in a vacuum oven at 80 °C. The product was labeled as 0-Cu_2_O/PP. In addition, for comparison, the M-Cu_2_O added to the PP matrix was prepared with the same parameters, and the product was labeled as M-Cu_2_O/PP.

### 2.5. Characterization Methods

Characterization of materials (Cu_2_O, Cu_2_O/PP composites) was done with X-ray diffraction (XRD), Fourier transform infrared (FTIR) spectroscopy, scanning electron microscopy (SEM) and energy dispersive X-ray spectroscopy (EDS) techniques. The structure of the prepared powders was characterized by X-ray diffraction (XRD) with Cu K_α_ radiation (λ = 1.5418 Å) (Philips, PANalytical X’pert).

Various functional groups present within Cu_2_O, KH570-Cu_2_O and KH-570 were identified by FTIR spectra by using an Interspec 2020-Spectrolab FTIR spectrometer (Thermo Fisher Scientific, Waltham, MA, USA) and KBr as reference. SEM coupled with EDS was used to study the surface morphology and chemical composition of the samples by using scanning electron microscope (Hirachi, Hitachi-S4800, Tokyo, Japan). EDS is an analytical technique used for the elemental analysis or chemical characterization of the materials. The thermal stability of Cu_2_O, KH570-Cu_2_O and Cu_2_O/PP composites was investigated by thermogravimetric analysis (TGA, Netzsch, TG209F3, Germany). The heating rate was 10 °C min^−1^, and the experiments were performed in a continuous air flow rate of 20 cm^3^ min^−1^. The thermal properties of the Cu_2_O/PP composites were determined by using differential scanning calorimetry (DSC, Netzsch, DSC214, Germany). For each test, the sample was first heated from room temperature to 230 °C with a heating rate of 10 °C/min^−1^ and annealed for 5 min to remove thermal history, followed by cooling down to room temperature at a rate of 10 °C/min^−1^ for data collection. Experiments were run on samples of about 6–8 mg.

## 3. Results and Discussion

### 3.1. XRD and SEM of As-Synthesized Cu_2_O and M-Cu_2_O

**Figure 1:**
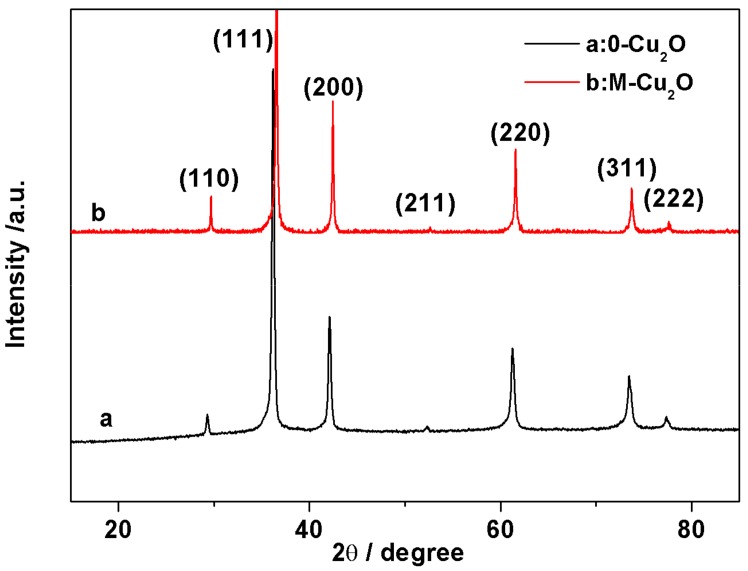
Powder X-ray diffraction (XRD) pattern of the 0-Cu_2_O and M-Cu_2_O.

Figure 1 shows typical XRD patterns of the synthesized-Cu_2_O and M-Cu_2_O. All the diffraction peaks of 0-Cu_2_O and M-Cu_2_O can be indexed to pure cubic phase Cu_2_O, indicating the cubic phase Cu_2_O was not affected by surface treatment. There are seven peaks with 2θ values of 29.58, 36.48, 42.39, 52.61, 61.52, 73.71 and 77.59, corresponding to (110), (111), (200), (211), (220), (311) and (222) crystal planes of Cu_2_O with cubic structure, respectively (standard JCPDS file no. 99-0041). The lattice constant calculated from this pattern was 4.2623 Å, which agrees with the standard value 4.269 Å. In addition, the XRD pattern of the as-synthesized powders in Figure 1 shows the sharp peak shape, indicating that the powders obtained by the current one-pot synthetic approach were well-crystallized. No characteristic peaks of CuO and Cu are observed in XRD patterns, indicating that high-purity Cu_2_O crystalline is successfully synthesized by this method. The pure crystal phase is attributed to the existence of glucose in the process of preparation. Because glucose is a weak reducing agent, only in a strongly basic solution can the Cu^2+^ be reduced to Cu^+^.

**Figure 2:**
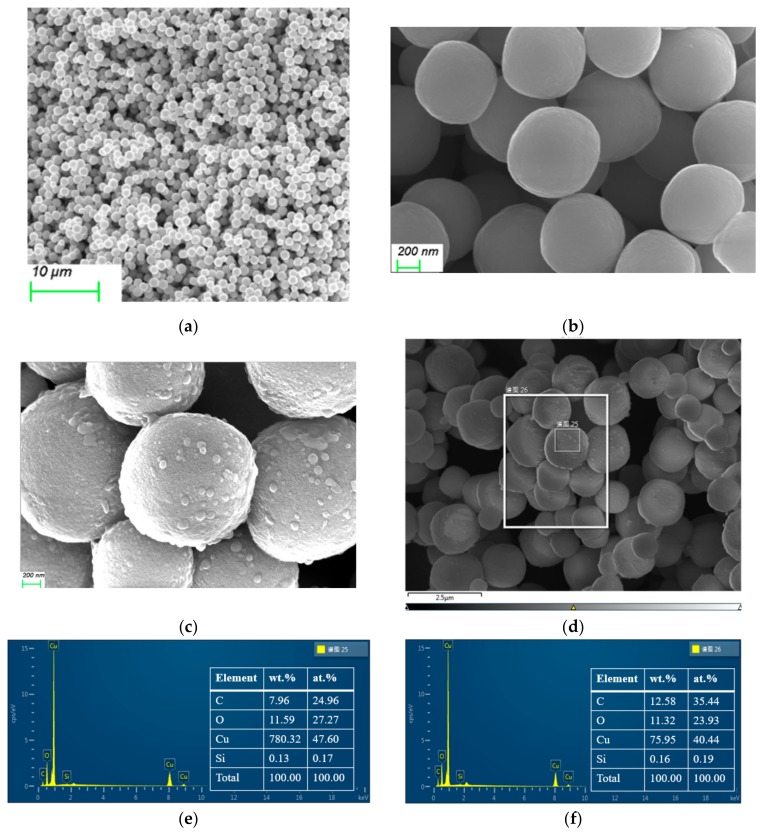
Scanning electron microscopy (SEM) image of (**a**,**b**) 0-Cu_2_O, (**c**) M-Cu_2_O, (**d**–**f**) EDS/SEM mapping for select area.

Figure 2a,b show the SEM images of as-synthetic Cu_2_O particles under different magnifications. It can be clearly seen that the powders have uniform, monodispersed and spherical particles with a mean diameter of 850 nm, because of having polyethylene glycol (PEG) as the surfactant. The steric hindrance effect of PEG can effectively prevent the agglomeration of nano Cu_2_O particles, thereby improving the uniformity of Cu_2_O particles. When the micrograph of the microsphere is further enlarged to high magnification (Figure 2b), it is found that the surface of the microsphere is very smooth, and this is different from other literature [44,45], in which the surface of the reported Cu_2_O particles is very rough, with small flakes and large particles of irregular morphology, or composed of abundant nanoparticles. Figure 2c–f give the SEM/EDS of M-Cu_2_O, and it can be seen that the surface of the M-Cu_2_O with rough surface is obviously uniformly covered by some substances. These substances are attached to the surface of the Cu_2_O sphere in granular form, indicating that the coupling process can be accomplished via the chemical reaction between the alkoxy group of the silane coupling agent and the hydroxyl groups (–OH) on the Cu_2_O surface (due to the hydroxyl groups (–OH) commonly existing on the surface of metal oxide that is used as a reaction site [46]), and a coupling agent layer is formed on the surface of Cu_2_O by a chemical bond.

With respect to the above as-synthesied Cu_2_O sphere, gelatin play important roles in the formation of this Cu_2_O sphere. The mechanism of formation of Cu_2_O the sphere was analyzed as follows. When the temperature is relatively high (50 °C), the gelatin sol can exist in the form of droplets in the solution. These droplets should serve as templates for the development of spherical structures. The gelatins contain large amounts of carboxyl and ammonia and the positive Cu^2+^ is easily absorbed on the surface of the gelatin droplets, due to electrostatic interaction. The reduction of Cu^2+^ by glucose makes Cu^+^ to nucleate and grow at the appropriate position.

### 3.2. Characterization of Surface Modification of Cuprous Oxide

#### 3.2.1. Fourier-Transform Infrared Spectroscopy (FTIR)

**Figure 3:**
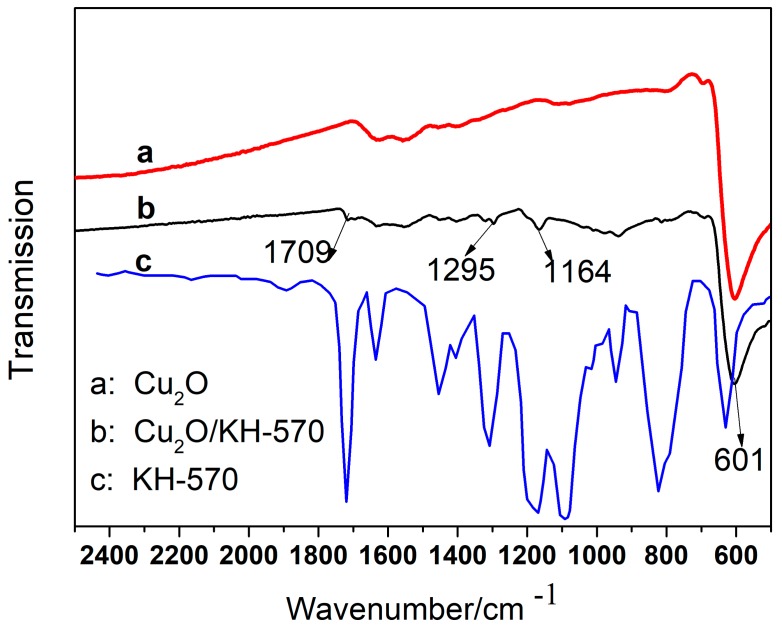
Fourier transform infrared spectroscopy (FTIR) spectra of (**a**) 0-Cu_2_O, (**b**) M-Cu_2_O, (**c**) KH-570.

NPs have a strong tendency to undergo agglomeration. Surface modification of the metal oxide NPs with physically (by physisorption) or chemically (through covalent bonding) routes have been employed to overcome this problem. Surface modification refers to the use of surface additives to change the surface state of particles, such as the surface atomic layer structure and functional groups, surface hydrophobicity, electrical properties, chemical adsorption and reaction characteristics. Through surface modification, it can improve the dispersibility, durability and weatherability of the powders, and improve also the surface activity. Thus, new physical and chemical properties can be produced on the surface of the particles. Surface modification of NPs is that a chemical reaction occurs on their surface, which introduces some new interaction forces between the two phases, such as van der Waals forces, hydrogen bonds or coordination bond interactions, and the combinations of ionic or covalent bonds [28]. The infrared spectrum of cuprous oxide before and after KH570 modification and KH570 is shown in Figure 3. The Figure 3a curve shows that the characteristic features in the FTIR spectrum of 0-Cu_2_O are the absorption bands corresponding to the Cu–O at 601 cm^−1^. After the Cu_2_O was modified by KH570, the three new characteristic features in the FTIR spectrum of M-Cu_2_O are the absorption bands corresponding to the α, β unsaturated esters –C=C–CO–OR– of KH570 at 1709 cm^−1^, the sp^2^ C–O stretching at 1295 cm^−1^, the sp^3^ C–O stretching at 1164 cm^−1^, respectively (Figure 3b). These three peaks prove the existence of the ester group after the reaction; that is, the purpose of surface modification of Cu_2_O by KH570 is achieved. 

#### 3.2.2. Thermogravimetric Analysis (TGA) of Inorganic Particles Cu_2_O

**Figure 4:**
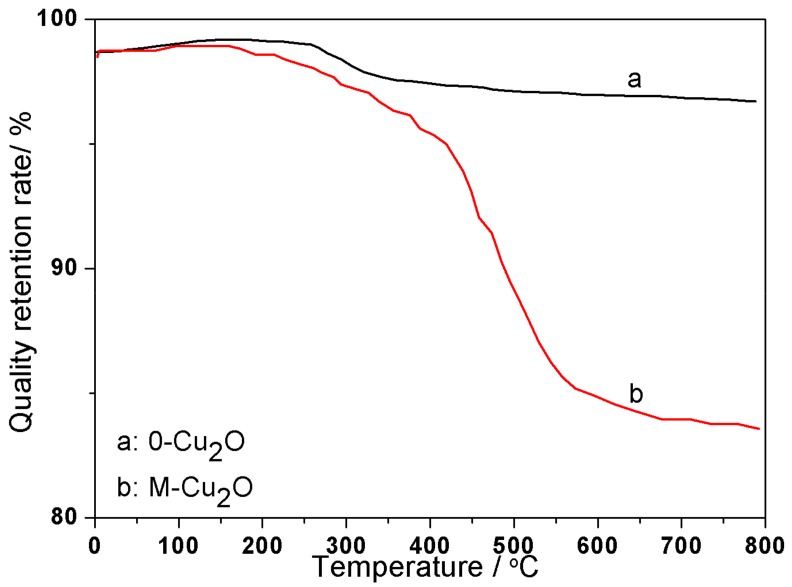
Thermogravimetric Analysis (TGA) of 0-Cu_2_O and M-Cu_2_O. (**a**) 0-Cu_2_O; (**b**) M-Cu_2_O.

Based on infrared spectroscopy, the thermogravimetric analysis (TGA) of the inorganic particles Cu_2_O before and after the modification was carried out. The results are shown in Figure 4. As shown in Figure 4, before 350 °C, the mass loss of the Cu_2_O and Cu_2_O/KH-570 is mainly due to the evaporation of water. After 350 °C, the mass of 0-Cu_2_O in the curve a has only a small amount of change, and the mass loss is due to the binding water between the molecules (Cu (OH)_2_ → -Cu-O-Cu + H_2_O). The quality of the M-Cu_2_O is greatly reduced after 350 °C, as shown in the Figure 4b curve. Combined with the infrared spectrum (Figure 3), a chemical bond forms between the silane coupling agent KH-570 and Cu_2_O, which is decomposed by heating, resulting in a rapid decrease in mass.

#### 3.2.3. Dispersion of 0-Cu_2_O and M-Cu_2_O in PP

**Figure 5:**
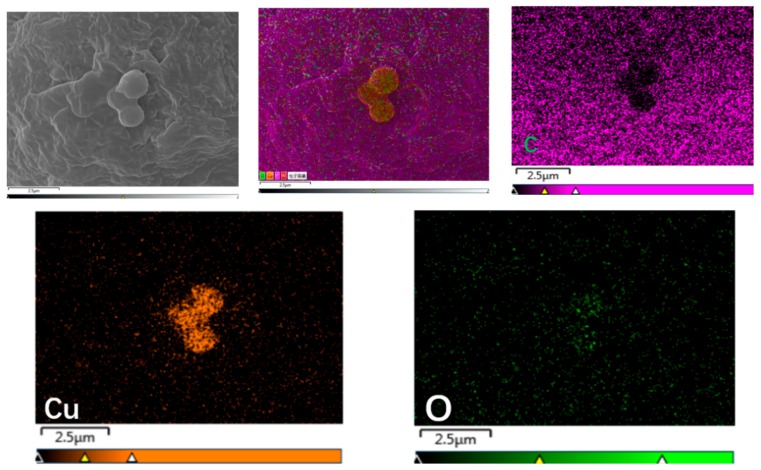
SEM images mapping of 0-Cu_2_O/PP composites, energy-dispersive X-ray spectroscopy (EDS)/SEM mapping for elements C, Cu and O.

**Figure 6:**
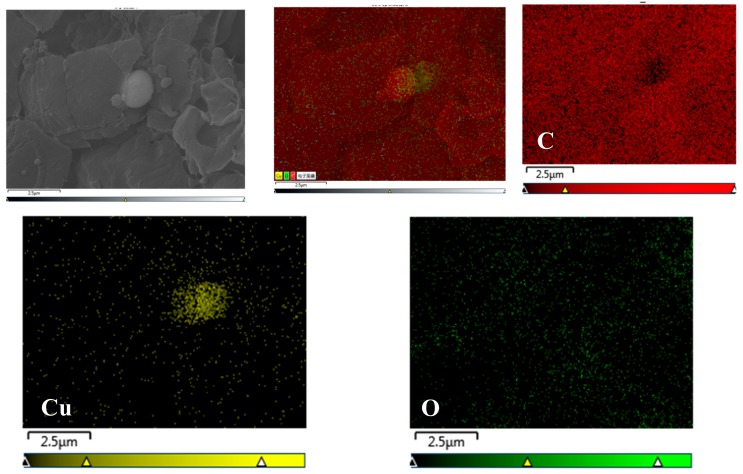
SEM images mapping of M-Cu_2_O/PP composites, EDS/SEM mapping for elements C, Cu and O.

The scanning electron microscopy (SEM) and energy-dispersive X-ray spectroscopy (EDS) of 0-Cu_2_O/PP and M-Cu_2_O/PP are given in Figure 5 and Figure 6. As shown in Figure 5 and Figure 6, the agglomerates of unmodified Cu_2_O in the PP matrix were found, but the KH570-modified Cu_2_O was finely dispersed in the PP matrix, without agglomeration. These results illustrate that unmodified inorganic superfine cuprous oxide has poor surface activity, resulting in aggregation, but the surface of superfine Cu_2_O were modified with KH570 to introduce organic functional groups (such as the ester group) on the surface of Cu_2_O (Figure 3), and improve the compatibility of the Cu_2_O surface (hydrophilic) with a hydrophobic polymer surface. This also increases the dispersion stability in organic media [47]. These results also reveal that the surface of Cu_2_O was successfully modified by the silane coupling agent KH-570.

### 3.3. Thermal Properties of 0-Cu_2_O/PP and M-Cu_2_/PP Composites

**Figure 7:**
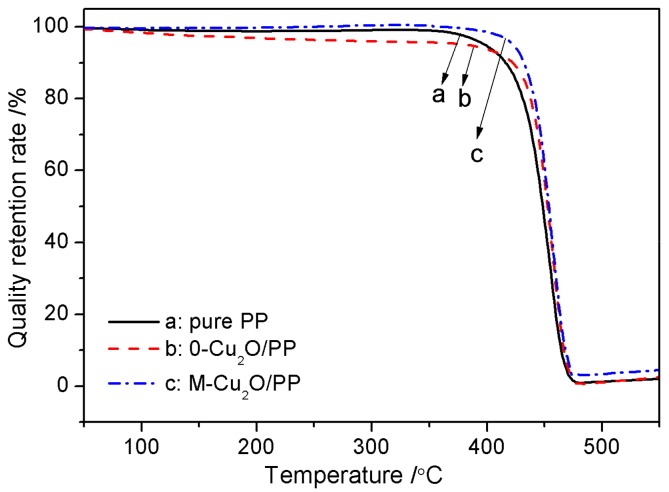
TGA of the Cu_2_O/PP composite.

Polypropylene (PP) is one of the five general-purpose plastics. It is cheap, non-toxic and tasteless, and has excellent mechanical properties. It is widely used in electrical appliances, automobiles, daily necessities, etc. [1,2,3,4], but its impact toughness is poor, its thermal stability is also poor, and it is easy to burn. Disadvantages greatly limit the application of PP in certain engineering fields. In order to broaden the application range of PP, the modification of PP has become a research hotspot. Making composite materials is a good way. Adding Cu_2_O to the PP matrix can improve the heat resistance of the composite. The TGA curve of Cu_2_O/PP at the rate of 10 °C/min was shown in Figure 7. As can be seen from Figure 7, the T_−5%_ (temperature of weightlessness 5%) values of pure PP, 0-Cu_2_O/PP and M-Cu_2_O/PP from the TGA curve were 398.6 °C, 383.4 °C and 422.8 °C, respectively. The T_−__5%_ value of 0-Cu_2_O/PP was lower than the value of pure PP, due to the evaporation of bound water between Cu(OH)_2_ molecules, as shown in Figure 4. The T_−__5%_ value of M-Cu_2_O/PP was higher than the value of pure PP and 0-Cu_2_O/PP, because the bonding force between Cu_2_O and PP was improved, after the Cu_2_O was modified by the silane coupling agent KH570. In the T_−__7%_ (temperature of weightlessness 7%) –T_−100%_ (temperature of weightlessness 100%) range of M-Cu_2_O/PP and 0-Cu_2_O/PP composites are higher than that of pure PP. The T_−__7%_ values of M-Cu_2_O/PP, 0-Cu_2_O/PP and pure PP were 432.1 °C, 422.3 °C and 416.3 °C, respectively. The T_max_ (temperature of the maximum weight loss rate) values of M-Cu_2_O/PP, 0-Cu_2_O/PP and pure PP were 485.2 °C, 483.4 °C and 479.6 °C, respectively. Obviously, the addition of Cu_2_O significantly improved the thermal stability of the PP matrix, due to the strong interaction between superfine Cu_2_O and the PP chain, which hinders the rupture of this PP chain. Combining Figure 4 and Figure 7, these results indicate that the thermal stability of PP can improve by adding inorganic metal oxide Cu_2_O, especially modified-Cu_2_O. Therefore, based on the potential bacteriostasis of cuprous oxide, the low cost of PP, and the results of this study, it is predicted that Cu_2_O/PP composites can be used in infant preparation (such as in milk bottles) with low cost and good thermal stability in the near future.

**Figure 8:**
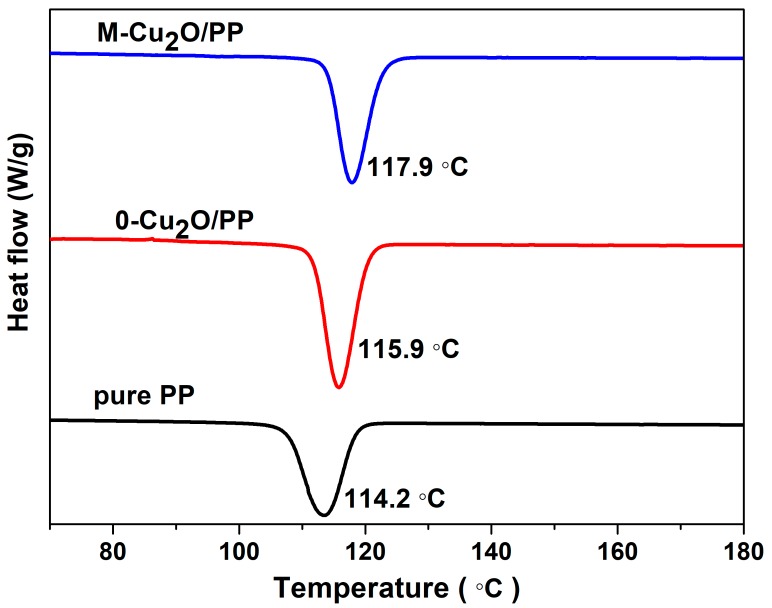
Cooling curves of pure polypropylene (PP) and Cu_2_O/PP composites.

**Figure 9:**
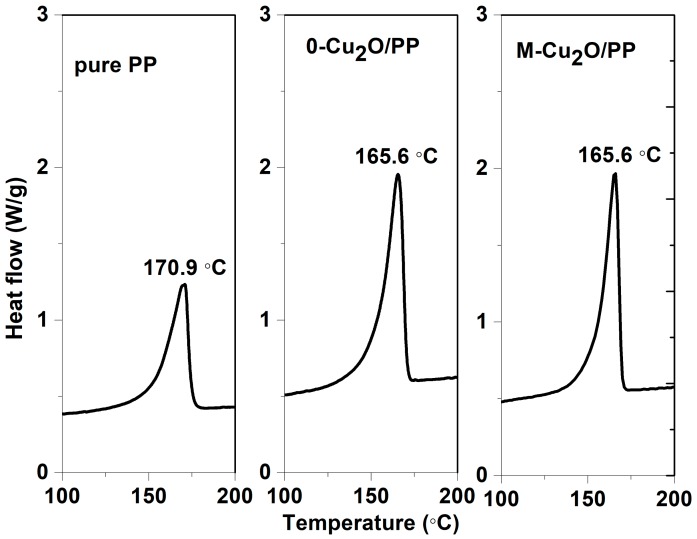
Melting curves for pure PP and Cu_2_O/PP composites.

Figure 8 and Figure 9 show the DSC curves for pure PP and Cu_2_O/PP composites. The crystallizing temperature of PP is increased by approximately 3 °C, when Cu_2_O is added to the PP. However, our results show that an increase of 3 °C in the crystallization temperature is achieved, implying that the Cu_2_O is a nucleating agent with a rather low efficiency, comparable with other widely published sources [48,49,50,51]. It was also found that the melting temperature of PP is higher than that observed for the Cu_2_O/PP composites.

## 4. Conclusions

Superfine cuprous oxide (Cu_2_O) spheres with a mean diameter of 850 nm have been synthesized by solution reduction. The surface of M-Cu_2_O is rougher than that of 0-Cu_2_O, and the Cu_2_O/PP composite was prepared by using melting blend. Fourier-transform infrared spectroscopy and the TGA curve revealed that the Cu_2_O had been successfully modified by silane coupling agent KH570. The KH570-modifed Cu_2_O was finely dispersed in the PP matrix from the SEM images, indicating that the modified cuprous oxide with active functional group, such as an ester group, can improve its compatibility with PP. The thermal stability of Cu_2_O/PP composites is better than that of pure PP, especially modified Cu_2_O/PP composites. Therefore, based on the potential bacteriostasis of cuprous oxide, the low cost of PP, and the results of this study, it is predicted that Cu_2_O/PP composites can be used in infant preparation (such as milk bottles) with low cost and good thermal stability in the near future.
